# Point-of-care brain magnetic resonance imaging in children with head trauma (BRAINCHILD): Protocol for a prospective observational cohort study

**DOI:** 10.1371/journal.pone.0346502

**Published:** 2026-04-13

**Authors:** Pradip P. Chaudhari, Kayla Guzman, Jason M. Toliao, Samantha Lozano, Christina Lee, Payal Shah, Frank Gonzalez, Bradley J. De Souza, Ryan Spurrier, Peter A. Chiarelli, Jonathan D. Santoro, Ramón Durazo-Arvizu, Eamon K. Doyle, Chia-Shang J. Liu, Marvin D. Nelson, Natasha Leporé

**Affiliations:** 1 Division of Emergency and Transport Medicine, Department of Pediatrics, Children’s Hospital Los Angeles, Los Angeles, California, United States of America; 2 Keck School of Medicine of the University of Southern California, Los Angeles, California, United States of America; 3 Children’s Hospital Los Angeles, Los Angeles, California, United States of America; 4 CIBORG Laboratory, Department of Radiology, Children’s Hospital Los Angeles, Los Angeles, California, United States of America; 5 University of Southern California, Los Angeles, California, United States of America; 6 Division of Critical Care Medicine, Department of Anesthesia Critical Care Medicine, Children’s Hospital Los Angeles, Los Angeles, California, United States of America; 7 Division of Pediatric Surgery, Department of Surgery, Children’s Hospital Los Angeles, Los Angeles, California, United States of America; 8 Division of Neurosurgery, Phoenix Children’s Hospital, Phoenix, Arizona, United States of America; 9 Division of Neurology, Department of Pediatrics, Children’s Hospital Los Angeles, Los Angeles, California, United States of America; 10 Biostatistics, Informatics and Data Management Core, Department of Pediatrics and Neurology, Children’s Hospital Los Angeles, Los Angeles, California, United States of America; 11 Department of Radiology, Children’s Hospital Los Angeles, Los Angeles, California, United States of America; 12 Departments of Radiology and Biomedical Engineering, University of Southern California, Los Angeles, California, United States of America; PLOS: Public Library of Science, UNITED KINGDOM OF GREAT BRITAIN AND NORTHERN IRELAND

## Abstract

**Background:**

Traumatic brain injury (TBI) is a leading cause of disability and death in children. More than 30% of children presenting to the emergency department (ED) with head trauma undergo brain computed tomography (CT), the standard neuroimaging modality in acute evaluation of intracranial pathology. Conventional magnetic resonance imaging (MRI) provides a non-ionizing alternative with greater sensitivity for certain intracranial injuries but is infrequently used in acute TBI because of limited scanner access and longer scan duration. Rapid brain MRI protocols reduce scan time and can be completed without sedation, with diagnostic accuracy for TBI comparable to CT, yet real-world availability remains limited. Point-of-care, low-field MRI (POC LF-MRI) systems are a recent radiologic advance that are portable, require less infrastructure, and allow bedside neuroimaging, including in critically injured children who cannot be safely transported. However, critical knowledge gaps exist regarding the diagnostic accuracy and feasibility of POC LF-MRI for pediatric head trauma in emergency and critical care settings.

**Objective:**

Our research aims to (1) determine the accuracy of POC LF-MRI for neuroradiographic TBI and clinically important TBI compared to current clinical standard of care initial neuroimaging, (2) determine the accuracy of POC LF-MRI for neuroradiographic injury progression on repeat neuroimaging, and (3) determine feasibility metrics and balancing measures of POC LF-MRI, including order-to-scan time, scan duration, proportion of incomplete scans, and ED length-of-stay.

**Methods:**

We will conduct a prospective, single-center, observational diagnostic accuracy cohort study of children 7–17 years old with blunt head trauma who undergo standard-of-care neuroimaging. Children with MR-unsafe implants or metallic shrapnel and wards of the state will be excluded. POC LF-MRI will be obtained within a reasonable time window of clinical neuroimaging, with a flexible window up to 72 hours, either in the ED, inpatient unit, or intensive care unit (ICU). The primary outcome is neuroradiographic TBI, defined as any traumatic intracranial finding on neuroimaging. Secondary outcomes include clinically important TBI (defined as TBI-related neurosurgical intervention, endotracheal intubation >24 hours, death, or ≥2-night hospitalization) and neuroradiographic injury progression on repeat neuroimaging (yes/no). Feasibility outcomes include order-to-scan time, scan duration, proportion of incomplete scans, and ED length of stay, along with other operational and balancing measures. Accuracy will be determined using imaging-level analyses comparing POC LF-MRI with clinical standard-of-care neuroimaging, reporting sensitivity, specificity, predictive values, and likelihood ratios with 95% confidence intervals for neuroradiographic TBI, clinically important TBI, and neuroradiographic injury progression, including predefined non-inferiority criteria for sensitivity, subgroup analyses, descriptive analyses of feasibility metrics, and exploratory analyses addressing incomplete imaging and missing data.

**Results:**

The project was funded in 2024, and enrollment will be completed in July 2026. Data analyses are expected to be completed by December 2026, and the primary study results will be submitted for publication in 2027.

**Conclusions:**

This study will evaluate accuracy and feasibility for POC LF-MRI in an important subset of pediatric trauma patients and will provide preliminary data to inform future multicenter studies evaluating POC LF-MRI for children with head trauma.

## Introduction

Head trauma is a leading cause of morbidity and mortality in children, accounting for more than 800,000 emergency department (ED) visits, 60,000 hospitalizations, and 6,000 deaths in the U.S. annually [1]. In injured children with head trauma, identification of traumatic brain injury (TBI) on neuroimaging is essential for optimal care. As a result, over 30% of children seen in the ED with head trauma undergo computed tomography (CT) annually [2]. When a child presents with head trauma, clinicians assess presenting signs and symptoms to establish risk for TBI and need for CT [3]. CT is used to establish an initial diagnosis of neuroradiographic TBI (e.g., imaging evidence of fracture, hemorrhage, edema) and serial CTs often are performed while monitoring for potential injury progression [4–14]. Although neuroimaging is frequently necessary, CTs carry the disadvantage of ionizing radiation exposure to the developing brain. The associated future cancer risk increases with radiation dose-per-scan and number of scans [15,16]. When evaluating a child with head trauma, clinicians must weigh the short-term risks of TBI against lifetime cancer risk from CT radiation. To improve this risk assessment, clinicians stratify children for head injuries that are the most clinically meaningful, which is termed *clinically important TBI* (ciTBI) [3,17,18]. ciTBI is a well-validated outcome used in clinical practice and for research, and is defined as neuroradiographic TBI with related neurosurgical intervention, endotracheal intubation, prolonged hospitalization, or mortality [3]. Even with appropriate risk stratification, a majority of clinically motivated CTs are negative for neuroradiographic TBI [3]. Similarly, in children with neuroradiographic TBI, the majority of follow-up CTs are negative for neuroradiographic progression [4–14].

While conventional magnetic resonance imaging (MRI) provides a radiation-free alternative with greater sensitivity for certain intracranial injuries than CT, its routine acute use is limited by scanner availability, longer exam durations, and the frequent need for sedation in younger children. Modern protocols for rapid conventional brain MRI reduce scan times and can be completed without sedation, with comparable accuracy to CT for neuroradiographic TBI and ciTBI [19–23]. However, widespread adoption is hindered by limited access to conventional MRI in emergency and critical care settings. Because neuroimaging is a near-essential diagnostic test for TBI, innovative approaches that enhance accessibility and safety while reducing radiation exposure can improve care delivery.

To overcome barriers associated with conventional MRI, point-of-care low-field MRI (POC LF-MRI) systems have been developed. These systems combine images acquired at low magnetic field strength (0.064 Tesla (T)) with automated imaging optimization to generate clinically useful images [24,25]. The system is substantially less expensive than conventional MRI, has fewer safety challenges compared to conventional MRI, is able to be deployed at bedside, can be operated by non-radiology technicians, and is Food and Drug Administration (FDA)-cleared for all age groups. Bringing neuroimaging to the bedside can be of substantial benefit to patients, particularly those who are too critically injured to be safely transported to a distant scanner [26]. Critical knowledge gaps remain, related to accuracy and feasibility of using POC LF-MRI for the emergency and critical care of children with head trauma. For this study, we plan to study pediatric patients 7–17 years old who can tolerate MRI completion without sedation to improve feasibility, with future work aimed to investigate younger age groups. Through a prospective, single-center, observational cohort study, we will evaluate the diagnostic accuracy and operational practicality of deploying POC LF-MRI for children with head trauma.

The objectives of the current study are to determine the accuracy of POC LF-MRI for TBI compared to current clinical standard of care initial neuroimaging, (2) determine the accuracy of POC LF-MRI for neuroradiographic injury progression on repeat neuroimaging, and (3) determine feasibility metrics and balancing measures of POC LF-MRI, including order-to-scan time, scan duration, proportion of incomplete scans, and ED length-of-stay.

## Materials and methods

### Study design and setting

This is a prospective, single-center, observational, diagnostic accuracy cohort study comparing POC LF-MRI to standard clinical neuroimaging. The study will be conducted at a large, freestanding tertiary pediatric Level 1 trauma center with a high-volume ED (annual visits ~98,000) and dedicated pediatric intensive care services. Children will be enrolled from the ED, inpatient units, and ICU.

### Study population

Eligible children will include those 7–17 years old with blunt head trauma who undergo standard-of-care neuroimaging with brain CT or MRI. We will exclude those with MR unsafe implants or metallic shrapnel. We selected 7 years as the lower age cutoff to improve feasibility based on the developmental stage at which children generally can remain still enough for MRI completion, eliminating the need for sedation, while supporting the testing of younger children in future work.

### Neuroimaging modalities

Clinical standard-of-care neuroimaging (head CT, conventional MRI, or rapid conventional MRI) will be obtained during routine clinical care at the discretion of the treating clinician’s discretion. No additional sedation or intravenous contrast will be administered for the purposes of the study. If sedation or contrast is administered for clinical care, it will not be altered for research purposes.

Head CT imaging will be performed using pediatric-specific protocols in accordance with institutional standards, and in line with efforts to minimize radiation exposure consistent with the ALARA (as low as reasonably achievable) principle. Conventional full- and rapid-protocol MRI studies will be performed using typical high-field (1.5 and 3 T) MRI systems according to standard institutional practices. All clinical neuroimaging studies will be interpreted by board-certified pediatric neuroradiologists as part of routine care. Final attending radiology reports from acquired clinical neuroimaging will serve as a comparator for determination of neuroradiographic and clinical outcomes.

At the study institution, conventional MRI is typically performed for patients with head trauma on fixed, 1.5T and 3T high-field MRI systems and is most commonly used for scheduled or non-emergent neuroimaging, including follow-up of known injuries. Conventional MRI is not routinely used for initial neuroimaging in pediatric head trauma because of limited scanner availability, longer scan duration, and challenges integrating MRI into emergency and critical care workflows. In younger children, longer scan duration frequently requires sedation, further limiting use in the acute setting. When obtained as part of clinical care, conventional full- or rapid-protocol MRI will be included as part of clinical comparator imaging for accuracy and feasibility analyses. Standard sequences in full-protocol MRI obtained for TBI include T1- and T2-weighted imaging, fluid-attenuated inversion recovery (FLAIR), diffusion-weighted imaging (DWI), and susceptibility weighted imaging (SWI) (<40 minutes total acquisition time). Anesthesia is not required for children able to remain still for full-sequence imaging (typically ≥7 years old).

Rapid-protocol conventional brain MRI utilizes an abbreviated number of sequences, designed to reduce scan duration to approximately 5 minutes. In pediatric trauma care, rapid MRI is most commonly used at the study institution for repeat neuroimaging in clinically stable children with known TBI. Despite shorter scan times compared with conventional MRI, real-world availability of rapid MRI varies based on scanner access, protocol availability, staffing, and clinical prioritization. When obtained as part of routine clinical care, rapid MRI studies will be interpreted by pediatric neuroradiologists and included as part of the clinical comparator neuroimaging for analyses of diagnostic accuracy, neuroradiographic injury progression, and feasibility metrics.

POC LF-MRI will be performed using the Hyperfine Swoop^®^ Portable MR Imaging^®^ system, which is a 0.064T portable brain scanner. Images are processed using system-standard automated imaging optimization algorithms to generate clinically interpretable images. The POC LF-MRI is an FDA-cleared Class II, non-significant risk device approved for all ages. Imaging sequences currently available on this platform include T1-weighted (Standard and Gray/White), T2-weighted (Standard and Fast), FLAIR, and DWI. POC LF-MRI will be obtained as close as feasible in time to clinical standard-of-care neuroimaging (goal target time within 12–24 hours of initial imaging), with a flexible predefined imaging window of up to 72 hours to allow for feasibility of enrollment integration within clinical workflow. POC LF-MRI findings will be compared with the clinical neuroimaging modality obtained in closest time proximity (CT, conventional MRI, or rapid MRI) to assess diagnostic accuracy, neuroradiographic injury progression, and feasibility outcomes, with additional analyses performed across modalities for patients who underwent multiple clinical neuroimaging modalities.

### Study procedures

Research staff will prospectively screen and identify eligible children during their routine clinical care for blunt head trauma. All clinical evaluation, management, and decisions regarding neuroimaging will be determined by the treating clinicians and will not be influenced by study participation. Following informed consent and assent, enrolled participants will undergo POC LF-MRI in close temporal proximity to clinical standard-of-care neuroimaging.

Personnel trained to use POC LF-MRI will image patients at the bedside. Enrolled children will undergo POC LF-MRI T2 Fast imaging (<3 minutes) in axial/coronal/sagittal planes. As tolerated, additional POC LF-MRI full-protocol scans will be obtained for analyses, including standard T2- and T1-weighted images, FLAIR, and DWI (total ~35 minutes). MRI technologists at the study institution are able to complete conventional MRIs in developmentally appropriate children ≥7 years old without sedation and will train research staff on child-friendly techniques to improve POC LF-MRI scan completion rates. Timing of imaging, scan duration, and completeness of imaging will be recorded for all participants.

Clinical data will be collected prospectively and through electronic health record abstraction. Data elements will include demographics, mechanism of injury, clinical signs and symptoms, Glasgow Coma Scale scores, vital signs, timing of injury and imaging, and details of clinical standard-of-care neuroimaging. Data regarding neurosurgical intervention, airway management, patient disposition, and length-of-stay will be collected as clinical outcome measures. Neuroimaging reports from clinical standard-of-care CT/MRI and radiologist interpretation of POC LF-MRI will be recorded using standardized data collection instruments. Imaging timing and sequence completion will be documented to support diagnostic accuracy and feasibility analyses. Electronic health record demographic and clinical data from missed eligible patients not screened prospectively will be compared with those enrolled to assess for enrollment bias.

### Outcomes

The primary outcome is neuroradiographic TBI, defined as any traumatic intracranial finding on neuroimaging including hemorrhage/contusion, cerebral edema, traumatic infarction, diffuse axonal/shear injury, venous sinus thrombosis, diastasis of cranial sutures, pneumocephalus midline shift, and/or brain herniation, with or without skull fracture. Isolated non-depressed skull fractures without additional intracranial injuries, which are clinically non-actionable [27], will not meet criteria for this outcome.

Secondary outcomes include ciTBI [3,17,18], defined as TBI associated with one or more of the following: neurosurgical intervention, endotracheal intubation for greater than 24 hours, death, or hospitalization of two or more nights related to the head injury. An additional secondary outcome is neuroradiographic injury progression on repeat neuroimaging, defined as any increase in size or extent of traumatic intracranial findings on follow-up imaging compared with initial imaging. Feasibility outcomes will include order-to-scan time, scan duration, scan interruption, proportion of incomplete scans, and ED/hospital/ICU length-of-stay. Additional operational and balancing measures will be collected to compare POC LF-MRI with clinical standard-of-care imaging, recognizing that certain metrics will be modality-specific.

### Imaging interpretation

Clinical standard-of-care CT images will be interpreted by pediatric neuroradiologists as per routine clinical care, and final attending radiology reports will be used for outcome determination. POC LF-MRI images will be interpreted independently by 2 study neuroradiologists. Scans will be provided individually and in random order. Given the different appearances by modality, modality cannot feasibly be masked for the radiologist, although radiologists will be masked to clinical information. We will assess interrater reliability using Cohen’s κ statistic. Imaging interpretation will be performed using standardized data collection instruments.

To ascertain accuracy of POC LF-MRI for neuroradiographic injury progression, paired scans (e.g., initial and repeat) will be provided and the process repeated for presence or absence of progression on repeat neuroimaging. Most children who undergo repeat neuroimaging only receive one additional scan (e.g., an initial CT followed by a second CT). However, some children are reimaged more than once (e.g., initial CT, followed by a second CT, followed by a rapid MRI). Neuroradiographic progression will be determined by comparison of the two images that are temporally paired.

### Statistical analysis

For Aim 1, we will determine accuracy of POC LF-MRI for neuroradiographic TBI and ciTBI by determining presence of these binary outcomes as assessed by initial clinical neuroimaging. The unit of analysis will be ‘imaging performed’, rather than ‘patient’, as each set of POC LF-MRI research sequences will be compared with corresponding standard clinical sequences. We will report descriptive statistics and test performance characteristics (sensitivity, specificity, predictive values, and likelihood ratios, with 95% confidence intervals (CIs)). We will perform subgroup analyses of accuracy for (i) combinations of POC LF-MRI sequences determined post-hoc to provide adequate visualization of structural abnormalities, (ii) individual abnormalities that are clinically meaningful (e.g., epidural, hemorrhage, subdural hemorrhage, cortical contusion, subarachnoid hemorrhage, midline shift, etc.), (iii) ciTBI without ≥2-night hospitalization, (iv) by age, (v) by imaging timing in comparison to time of injury.

For Aim 2, we will determine accuracy of POC LF-MRI for neuroradiographic injury *progression* by determining presence of this binary outcome as assessed by review of a pair of clinical neuroimaging in children who undergo repeat neuroimaging, in the order they undergo the scans. Similar to Aim 1, the unit of analysis will be the imaging performed, rather than the patient, as each research imaging pair will be compared with a pair of clinical imaging scans. We will report descriptive statistics and test performance characteristics (sensitivity, specificity, predictive values, and likelihood ratios, with 95% CIs). We will perform subgroup analyses of the accuracy of POC LF-MRI for different variations of imaging modalities (CT, full-protocol conventional MRI, rapid-protocol conventional MRI).

For Aim 3, we will utilize descriptive statistics to analyze feasibility metrics and balancing measures for POC LF-MRI and standard clinical neuroimaging. As a narrative review, we will explore clinical and neuroradiographic findings for concordant and discordant imaging interpretations, including review of injuries identified on POC LF-MRI not seen on clinical neuroimaging. We will conduct subgroup analyses based on patient stability to account for differing clinical approaches and safety profiles of conventional imaging in stable versus unstable trauma patients. Modality-specific operational data (e.g., ED arrival to first image for standard-of-care scans) will be collected accordingly.

Based on our findings from prior work at the same institution, capture of relevant clinical variables is expected to be > 95%. If necessary, we will impute missing data using methods that account for random versus non-random missingness.

### Sample size estimates

This study is designed to generate preliminary data on the diagnostic accuracy and feasibility of POC LF-MRI for pediatric head trauma. Enrollment is planned over an 18-month period, with an anticipated sample size of approximately 117 children aged 7–17 years with blunt head trauma who undergo clinical standard-of-care neuroimaging. Based on local prevalence estimates, among children undergoing neuroimaging, approximately 20% are expected to have neuroradiographic TBI and approximately 8% are expected to have ciTBI. Among children with neuroradiographic TBI, approximately 55% are expected to undergo repeat neuroimaging, with neuroradiographic injury progression identified in an anticipated 22% of repeat studies. Using these assumptions, we estimate that the planned sample of 117 children will include approximately 47 children with neuroradiographic TBI (40.2%; 95% prevalence CI: 31.2, 49.6%), 18 with ciTBI (15.4%; 95% prevalence CI: 9.4, 23.2%), and 5 with neuroradiographic injury progression on repeat neuroimaging (4.3%; 95% prevalence CI: 1.4, 9.7%).

Sample size considerations for diagnostic accuracy analyses were based on feasibility of enrollment of eligible patients, estimating sensitivity for neuroradiographic TBI and ciTBI with adequate precision, and on testing non-inferiority of POC LF-MRI compared with clinical standard-of-care neuroimaging. Required sample sizes were estimated assuming a power of 80%, across a range of outcome prevalences (5–40%), assumed true sensitivity (ranging from 85% to 95%), reference sensitivity value (10% lower than true sensitivity [28–32]), and a non-inferiority constant of δ = −5%. The reference sensitivity value assumes that POC LF-MRI is at least as sensitive as this number (e.g., 75%, 80%, 85%, etc.). POC LF-MRI will be considered non-inferior to clinical neuroimaging if the lower limit of the one-sided 97.5% CI for the sensitivity difference is larger than the non-inferiority constant δ.

These estimates indicate that a sample size of approximately 78 children would be sufficient to test non-inferiority for neuroradiographic TBI if the true sensitivity of POC LF-MRI is at least 85% and outcome prevalence is approximately 40%. If the outcome prevalence is approximately 20%, an estimated sample size of approximately 98 children would be required if the true sensitivity is approximately 94% and approximately 83 children if sensitivity is approximately 95%. Given the planned enrollment of 117 children, this study is anticipated to have sufficient power to evaluate the primary diagnostic accuracy outcomes and to generate estimates of sensitivity for neuroradiographic TBI and ciTBI. These data will be used to refine prevalence assumptions, sensitivity estimates, and sample size calculations for a future multicenter validation study.

### Ethics approval

This study has been approved by the institutional review board of the study site (IRB number: 23–00235). Written informed consent and assent will be obtained in accordance with institutional and regulatory requirements. The study involves minimal risk to patients and families.

### Study status and timeline

Enrollment is planned over an 18-month period, with an anticipated sample size of approximately 117 children. Actual enrollment and feasibility of data collection will depend on real-world clinical factors, including patient stability, clinical workflow integration, and family willingness to participate during acute care for pediatric trauma, when children are injured and families may be under stress. Data collection and analysis will be conducted following completion of enrollment.

## Results

Patient enrollment began on August 1, 2024 and is estimated to be completed on July 31, 2026. Data clean-up and analysis is projected to be completed by December 2026, and the results are expected to be submitted for peer-reviewed publication in 2027.

## Discussion

TBI is a common and serious condition in childhood, for which neuroimaging is essential in acute evaluation and management [1,2,33]. In current practice, CT is the predominant imaging modality because of its speed and accessibility [2,3,17], despite exposing children to ionizing radiation [15,16] and yielding negative studies for neuroradiographic TBI in the majority of clinically motivated studies [3]. Similarly, most follow-up CTs performed in children with neuroradiographic TBI do not demonstrate neuroradiographic injury progression [4–14]. As a result, contemporary pediatric head trauma care relies on imaging strategies that prioritize short-term diagnostic certainty while contributing to a cumulative radiation burden at the population level, requiring clinicians to balance immediate clinical risk against longer-term radiation-associated harm.

MRI provides a non-ionizing radiation alternative to CT with greater sensitivity for certain traumatic intracranial injuries [21,34–36]. yet conventional MRI remain underutilized in acute pediatric head trauma because of persistent limitations in scanner access, scan duration, and integration into emergency and critical care workflows. Even in high-resource pediatric centers, availability of rapid or conventional MRI varies by staffing and time of day [37], and limiting consistent use as a CT alternative. These constraints underscore the opportunity for scalable neuroimaging approaches that maintain diagnostic accuracy while improving accessibility and safety in real-world trauma settings.

POC LF-MRI systems have been developed that have the potential to address several of these limitations by combining images acquired at low magnetic field strengths with automated imaging optimization to generate clinically useful images [24,25]. Bringing neuroimaging to the bedside may be particularly beneficial for critically injured children who are not safe candidates for transport to a distant scanner [26]. In considering the expanded use of POC LF-MRI, there are knowledge gaps related to diagnostic accuracy and feasibility for pediatric head trauma in emergency and critical care settings that require dedicated investigation.

The BRAINCHILD study was designed to address such knowledge gaps through a prospective, single-center, observational diagnostic accuracy cohort study of children with blunt head trauma. By comparing POC LF-MRI with the clinical neuroimaging modality obtained as part of routine care, including CT, full-protocol conventional MRI, and rapid-protocol conventional MRI, this study was designed to reflect real-world clinical practice. The use of clinically meaningful and well-validated outcomes, including neuroradiographic TBI, ciTBI, and neuroradiographic injury progression, aligns the study with existing pediatric head trauma research and supports interpretation in a clinical context [3,17,18]. In addition, the collection of feasibility metrics and balancing measures provides insight into operational considerations relevant to ED, inpatient, and ICU workflows.

This study has several strengths. The prospective design allows for standardized data collection and image interpretation, while minimizing disruption to clinical care. Image interpretation is performed by pediatric neuroradiologists using independent review with adjudication and assessment of interrater reliability. Inclusion of both initial and repeat scans allows evaluation of POC LF-MRI across multiple clinically relevant scenarios. Several study limitations exist. This is a single-center study conducted at a high-volume pediatric Level 1 trauma center, which may limit generalizability to other settings. Enrollment feasibility depends on real-world clinical factors, including patient stability, integration into clinical workflow, and family willingness to participate during acute hospitalization for pediatric trauma, when children are injured and families may be under significant stress. To improve feasibility, the study is limited to children aged 7–17 years who are able to tolerate MRI without sedation, with the exclusion of younger children. In addition, radiologists cannot be blinded to imaging modality due to inherent differences in image appearance, although they will be masked to clinical information. Given the study duration and sample size estimates, this work is not powered to definitively evaluate small differences in diagnostic performance or subgroup effects, and findings should be interpreted in the appropriate context of supplied confidence intervals.

The primary goal of this study is to generate high-quality data to inform current clinical practice and future research. If POC LF-MRI demonstrates acceptable diagnostic accuracy and feasibility in this cohort, these data will support the design of future multicenter validation studies and the evaluation of broader patient populations, including younger children. This work will also inform optimal clinical scenarios for POC LF-MRI use, recognizing that in its current form, POC LF-MRI may augment rather than replace existing clinical neuroimaging strategies. Given the expanding availability of low-field MRI technology, rigorous evaluation of accuracy, feasibility, and workflow integration is necessary to ensure appropriate and effective use in pediatric trauma care.
